# Dissecting the dual role of OTU family proteins in tumor progression and immune escape

**DOI:** 10.3389/fimmu.2025.1544341

**Published:** 2025-05-21

**Authors:** Xiaolong Tang, Yadan Li, Yongshuo Liu

**Affiliations:** ^1^ Department of Laboratory Medicine, Hospital of Chengdu University of Traditional Chinese Medicine, Chengdu, Sichuan, China; ^2^ Department of Clinical Laboratory, Binzhou Medical University Hospital, Binzhou, Shandong, China; ^3^ Department of Clinical Laboratory, Shandong Cancer Hospital and Institute, Shandong First Medical University and Shandong Academy of Medical Sciences, Jinan, Shandong, China

**Keywords:** OTU family, deubiquitinating enzymes, tumorigenesis, immune regulation, ubiquitination-deubiquitination balance

## Abstract

As a core mechanism regulating intracellular protein homeostasis, the dynamic equilibrium between ubiquitination and deubiquitination profoundly impacts the functionality and fate of target proteins. The Ovarian tumor domain (OTU) family, a vital subclass of deubiquitinating enzymes, comprises 16 members that mediate ubiquitin binding and hydrolysis through their characteristic OTU domain. Recent years have witnessed growing interest in OTU family members in oncology and immunology research. This review comprehensively elucidates the core mechanisms by which OTU members regulate tumor-associated signaling networks via substrate-specific deubiquitination. On one hand, they directly govern tumor cell proliferation, metastasis, and apoptosis by modulating the stability of key substrates. On the other hand, they orchestrate tumor progression through dynamic regulation of inflammatory intensity, immune response duration, and immune evasion mechanisms within the tumor microenvironment (TME), thereby constructing a multidimensional regulatory network in tumor development. These findings not only unveil the pivotal role of OTU family members in tumorigenesis and immune modulation but also establish a theoretical foundation for developing novel *anti*-tumor therapeutics targeting deubiquitination processes. Notably, OTUs emerge as high-potential therapeutic targets with high translational relevance for refining precision-guided tumor-immunotherapy integration strategies.

## Introduction

**Figure 1:**
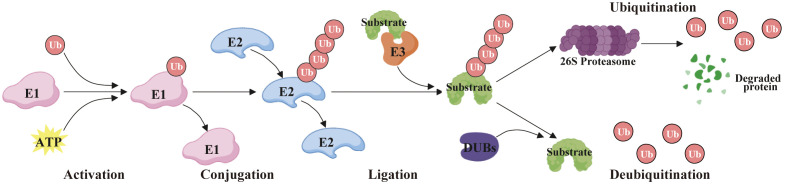
Diagram of ubiquitination and deubiquitination mechanisms.

Protein ubiquitination is a significant post-translational modification process that denotes the covalent attachment of ubiquitin to specific lysine residues of a target protein via an enzymatic reaction. During this procedure, the ubiquitin-activating enzyme (E1) first forms the ubiquitin-acyl acylase complex by activating the ubiquitin molecule. Second, the activated ubiquitin is transferred to the ubiquitin-conjugating enzyme (E2), forming the ubiquitin-E2 complex. Then, ubiquitin ligase (E3) is responsible for recognizing specific target proteins and transferring ubiquitin from E2 to lysine residues of the target protein. Ultimately, the ubiquitin on the target protein can further bind to other ubiquitin molecules to form polyubiquitin chains, which are usually a hallmark of signal transduction or degradation (1) (**Figure 1**). Protein ubiquitination is essential in protein degradation, signal transduction, DNA repair, and immune response, and the reason for its functional diversity is the variety of ubiquitination types, categorized as mono-ubiquitination and polyubiquitination. Generally, the polyubiquitin chains are formed by seven lysine residues (K6, K11, K27, K29, K33, K48, and K63) and one N-terminal Met1 (2). Of these, the K48 polyubiquitin chains (K48-Ub) are the most prevalent form, which usually symbolizes that the protein is about to be degraded by the proteasome, whereas the K63 polyubiquitin chains (K63-Ub) are typically implicated in signaling and DNA repair, etc., and is not directly engaged in the degradation of proteins (3). These various types of ubiquitination mechanisms permit the cell to precisely regulate protein function, reflecting the sophisticated intracellular regulatory network.

Deubiquitinases (DUBs) are a class of proteases that reverse protein ubiquitination by specifically removing ubiquitin molecules from substrate proteins, thereby regulating their stability, activity, and biological functions. As critical regulators of the ubiquitin-proteasome system (UPS), DUBs modulate cellular processes such as proliferation, differentiation, apoptosis, and stress responses through the removal or editing of ubiquitin chains. Dysregulation of DUB activity is closely associated with tumorigenesis and cancer progression (4). Based on their catalytic domain features, DUBs are categorized into five major families: Ubiquitin-specific proteases (USP), Ubiquitin C-terminal hydrolases (UCH), Machado-Joseph disease proteases (MJD), Ovarian tumor proteases (OTU), and JAMM/MPN metalloproteases. Members of these families exhibit marked functional heterogeneity in cancer contexts (5).

Taking OTUB1 (from the OTU family) and USP7 (from the USP family) as examples, their mechanisms in tumorigenesis and therapeutic targeting diverge significantly. OTUB1 drives malignancy by mediating tumor immune evasion, promoting cell migration, and regulating tumor grading-associated signaling pathways (e.g., in glioma) (6); USP7 predominantly facilitates oncogenesis by stabilizing oncoproteins (e.g., in hepatocellular carcinoma) (7), enhancing tumor cell proliferation (e.g., in non-small cell lung cancer) (8), and suppressing tumor suppressor activity (e.g., p53) (9). This functional divergence informs distinct therapeutic strategies: interventions targeting OTUB1 prioritize immunomodulation and migration inhibition, whereas USP7-focused therapies aim to restore tumor suppressor function and block pro-proliferative signaling (10).

To date, research on the OTU family is rapidly evolving, revealing significant potential in disease onset, progression, and treatment. Thus in this review, we describe comprehensively the roles of OTU family members in oncology and immunity, including the functions of OTUs in tumorigenesis, tumor stemness, ferroptosis, DNA repair, chemo- and radiotherapy, clinical relevance, inflammatory response, autoimmunity, *anti*-viral immunity, and *anti*-tumor immunity.

## Essential characteristics of the OTUs

**Figure 2:**
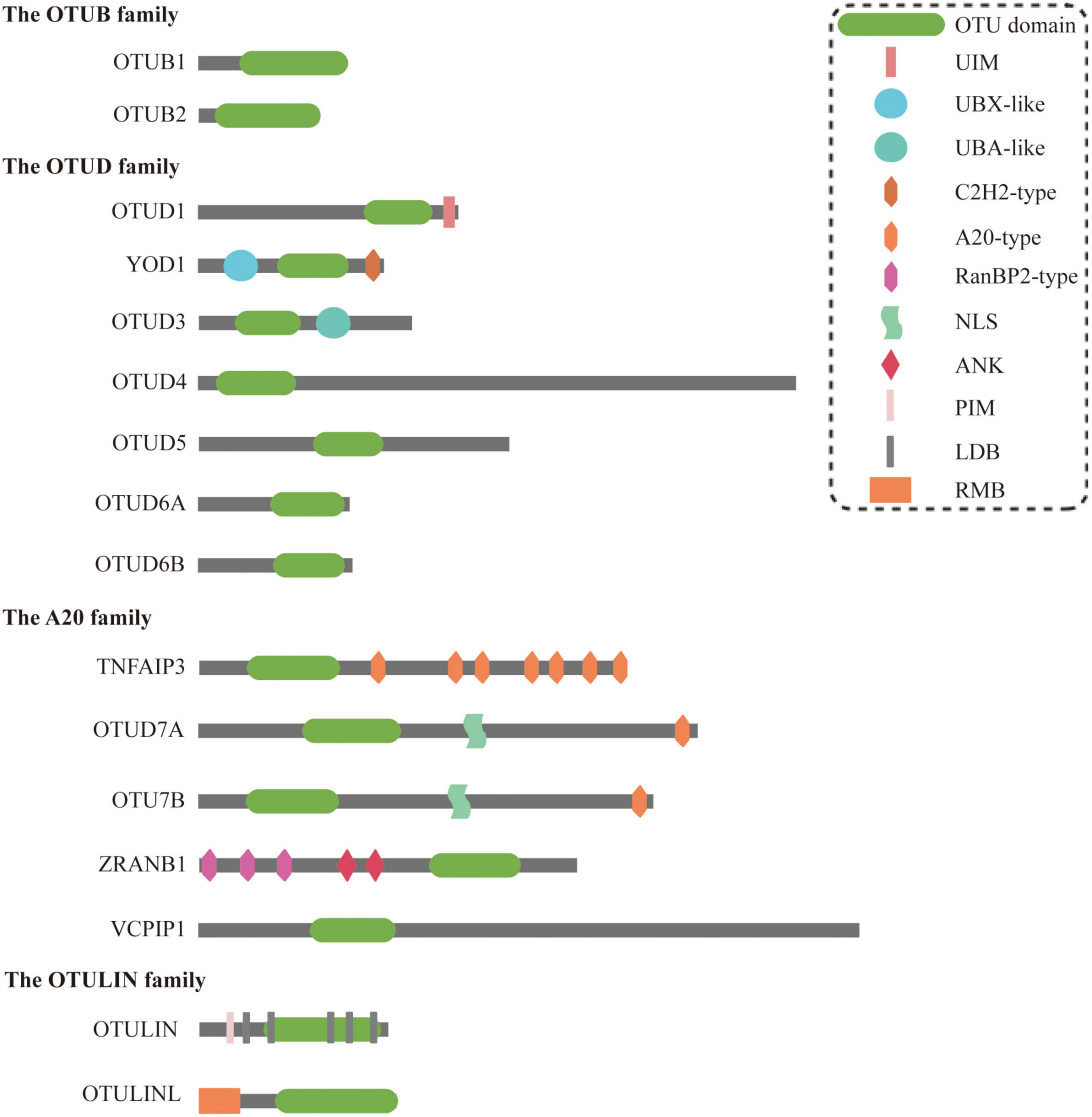
Structural characteristics of OTU family members. OTU domain, ovarian tumor domain; UIM, ubiquitin-interacting motif; UBX-like, ubiquitin regulatory X-like; UBA-like, ubiquitin-associated-like; NLS, nuclear localization signals; ANK, ankyrin motif; PIM, PUB interacting motif; LDB, linear diubiquitin binding; RMB, required for membrane binding.

The OTU family comprises DUBs that regulate protein stability and function by removing ubiquitin modifications. These enzymes are integral to diverse cellular processes, including signaling, cell cycle control, immune responses, and stress adaptation. To date, 16 OTU deubiquitylases have been identified, classified into four subfamilies: (a) the OTUB subfamily (OTUB1 and OTUB2); (b) the OTUD subfamily (OTUD1, YOD1/OTUD2, OTUD3, OTUD4, OTUD5/DUBA, OTUD6A, and OTUD6B); (c) the A20-like subfamily (TNFAIP3/A20, OTUD7A/Cezanne2, OTUD7B/Cezanne, ZRANB1/TRABID, and VCPIP1); (d) the OTULIN subfamily: OTULIN/FAM105B and OTULINL/FAM105A) (11–13). All members harbor a conserved OTU domain (OTUD) responsible for catalytic activity, although OTULINL lacks catalytic triad residues. Most OTU enzymes also contain auxiliary domains (e.g., UBDs) that refine substrate specificity (14) (**Figure 2**).

Interestingly, various OTU family members exhibit obvious disparities in specificity against the eight ubiquitin linkage types. At low concentrations, the six OTUs preferentially cleave only one ubiquitin chain (OTUD7A/OTUD7B-K11, OTUB1/OTUD4-K48, OTUD1-K63, and OTULIN-Met1); the four OTUs cleave two ubiquitin chains (OTUD3-K6/K11, TNFAIP3/VCPIP-K11/K48, and phosphorylated OTUD5-K48/K63); and four OTUs (OTUB2, OTUD2, OTUD6A, and ZRANB1) cleaved three or more chains preferentially (14). Elevated enzyme concentrations expand substrate promiscuity, but Met1 linkage hydrolysis remains uniquely dependent on OTULIN (14). These activities enable OTUs to regulate both proteasomal degradation and non-degradative signaling, positioning them as critical players in tumorigenesis.

Members of the OTU family, particularly OTUB1, rely not only on their deubiquitinase activity but also engage in non-canonical mechanisms to participate in tumorigenesis and progression. For example, (a) Ubiquitin transfer blockade: In multiple myeloma, OTUB1 interacted with the E2 enzyme UBE2D3 to inhibit ubiquitination of the transcription factor c-Maf, stabilizing its expression and promoting tumor cell survival (15). (b) Direct target protein binding: OTUB1 suppressed ubiquitination of proteins (e.g., HIF-1α and RACK1) through non-catalytic binding, thereby driving tumor progression (16, 17). (c) Phosphorylation-dependent functional switching: Phosphorylation of the Tyr26 residue in OTUB1 enabled its interaction with the cell cycle regulator p27, modulating p27 stability and cell cycle progression (18). The non-canonical mechanisms of the OTU family expand the functional landscape of DUBs in cancer, offering novel avenues for the development of precision anticancer strategies.

## OTUs in tumorigenesis, progression and metastasis

**Figure 3:**
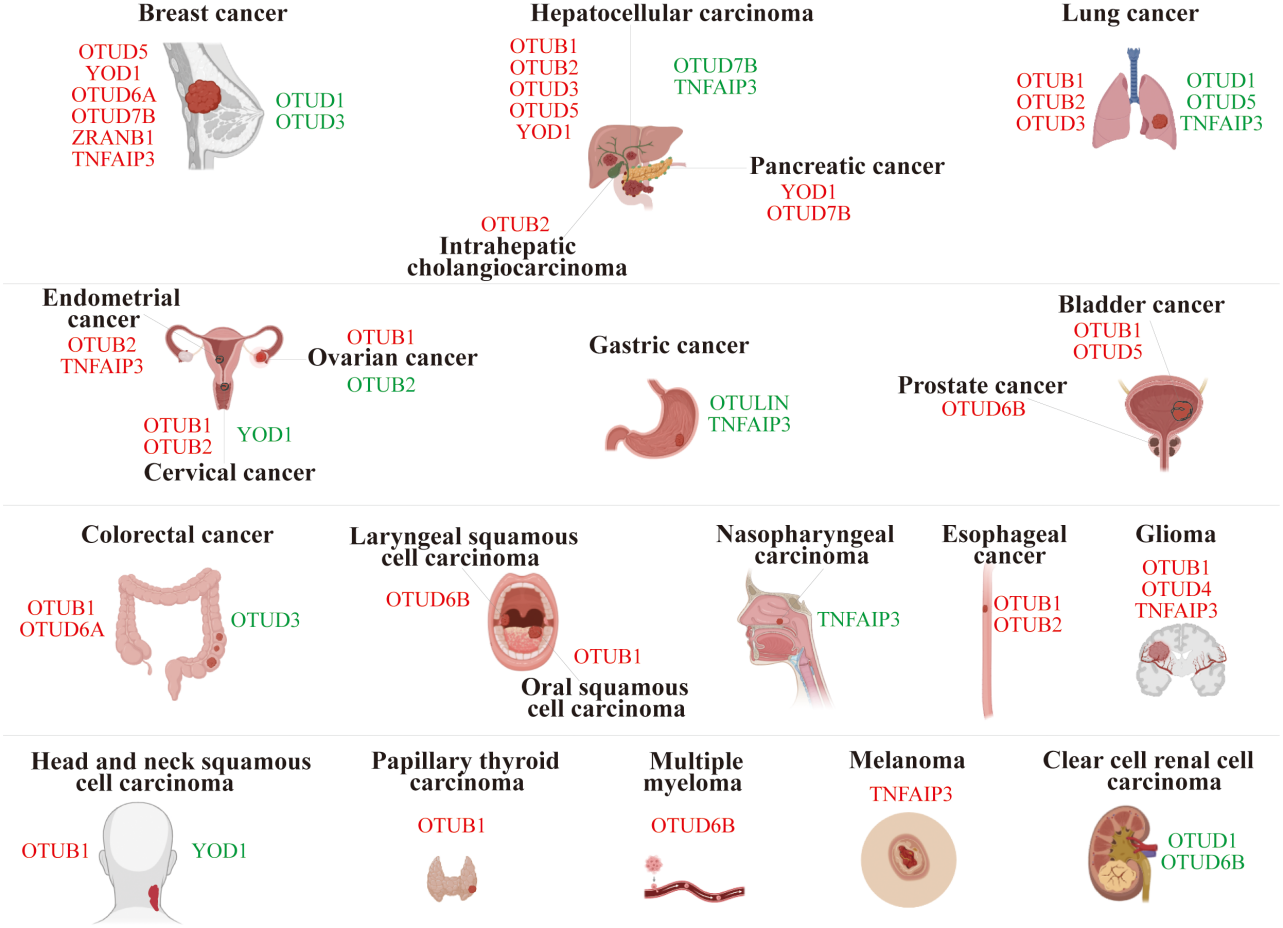
Role of OTU family members in tumor progression. The red font represents OTUs exerting an oncogenic effect in specific cancers, while the green font is for an inhibitory effect.

Members of the OTU superfamily are pivotal in carcinogenesis and progression, with their roles varying between oncogenic and tumor-suppressive capabilities depending on the type of cancer and the function of the substrate proteins (**Figure 3**).

### Breast cancer

In breast cancer (BC), OTUD5, YOD1, OTUD6A, OTUD7B, ZRANB1, and TNFAIP3 were characterized as carcinogenic drivers. Specifically, OTUD5 mediated the deubiquitination of Yes-associated protein (YAP), leading to increased YAP expression in THP-1-derived macrophages. Overexpression of YAP in M2 macrophages promoted triple-negative breast cancer (TNBC) progression both *in vitro* and *in vivo* (19). Similarly, YOD1 facilitated the deubiquitination of Cyclin dependent kinase 1 (CDK1), resulting in its upregulation, which enhanced the proliferation, migration, and invasion of TNBC cells (20). The study revealed that in breast cancer, OTUD6A stabilized DNA topoisomerase 2 binding protein 1 (TopBP1) by inhibiting its K48-Ub (21). OTUD7B’s deubiquitination and ubiquitin-binding functions enabled EGFR to evade cellular degradation (22), while ZRANB1 bound to and deubiquitinated EZH2, stabilizing it (23). Additionally, TNFAIP3 promoted epithelial-mesenchymal transition (EMT) in TGF-β1-induced breast cancer cells by facilitating multiple monoubiquitinations of Snail (24). Together, these processes enhanced cell proliferation, migration, and malignancy in breast lesions.

However, OTUD1 and OTUD3 could curb the progression of breast cancer. For example, OTUD1 weakened the tumor response to TGF-β by removing ubiquitin from SMAD7, thereby inhibiting breast cancer proliferation (25). OTUD3 effectively inhibited the proliferation and induced apoptosis of breast cancer cells by directly deubiquitinating and stabilizing p53 (26). Inversely, OTUD3 deficiency activated the AKT signaling pathway and propagated the transformation and metastasis of breast cancer cells (27).

### Hepatocellular carcinoma

In hepatocellular carcinoma (HCC), OTUB2, OTUD3, and OTUD5 stabilized the expression of PJA1 (28), ACTN4 (29), and SLC38A1 (30) by deubiquitination, respectively, which facilitated the proliferation and metastasis of HCC. Interestingly, OTUB1 reduced the K1-Ub of RACK48 through its non-classical inhibition of ubiquitination activity, thereby stabilizing RACK1 protein levels in HCC cells (17). YOD1, a key regulator of the Hippo pathway, stabilized ITCH and potentiated ITCH-mediated ubiquitination and degradation of LATS1/2, which resulted in elevated YAP/TAZ levels (31, 32). Both of these ultimately exacerbated the progression of HCC.

However, both OTUD7B and TNFAIP3 can exert *anti*-tumor effects by inhibiting the NF-κB signaling pathway in HCC (33, 34). Additionally, TNFAIP3 restrained the onset of EMT in HCC cells by diminishing Twist1 expression (34, 35).

### Lung cancer

In lung cancer, OTUB1 triggered lung cancer development by inhibiting RAS monoubiquitination (36); OTUB2 stabilized U2AF2 through the AKT/mTOR signaling pathway to promote the Warburg effect and tumorigenesis (37); and OTUD3 stabilized GRP78 to augment the malignancy (38). These play a crucial driving role in the progression of lung cancer.

Conversely, OTUD1 and OTUD5 deubiquitinated and stabilized KLF4, FHL1, and PTEN, respectively, which effectively suppressed the progression of non-small cell lung cancer (NSCLC) (39–41). Furthermore, silencing TNFAIP3 promoted lung cancer invasion and proliferation (42).

### Ovarian cancer

OTUB1 drove ovarian cancer (OV) progression by stabilizing FOXM1 via cleavage of K48-Ub of FOXM1 (43). Whereas OTUB2 acted as a tumor suppressor in OV, mechanistically, OTUB2 silencing destabilized SNX29P2, which subsequently prevented the degradation of HIF-1α. Elevated HIF-1α activated CA9 transcription and drove OV progression via promoting glycolysis (44).

### Bladder cancer

OTUB1 promoted bladder cancer (BLCA) progression by deubiquitinating and stabilizing ATF6 in response to endoplasmic reticulum stress (45). In addition, OTUD5 can activate the mTOR signaling pathway and facilitate BLCA progression. Mechanistically, OTUD5 deubiquitinated and stabilized RNF186, which further led to the degradation of sestrin2, an inhibitor of the mTOR signaling pathway (46).

### Endometrial cancer

OTUB2 contributed to endometrial cancer (EC) progression by regulating the PKM2-mediated PI3K/AKT signaling pathway (47). Furthermore, TNFAIP3 impeded ERα protein degradation through deubiquitinating enzyme activity, which enhanced estrogen-driven EC cell proliferation (48).

### Gastric cancer

Knockdown of OUTLIN, a gastric cancer (GC) biomarker, suppressed GC cell viability and metastasis (49). Chronic Helicobacter pylori infection, a major contributing factor to gastric carcinogenesis, induced TNFAIP3 to suppress caspase-8 activity through promoting K63-linked deubiquitination of procaspase-8 during infection, thereby reducing apoptotic cell death in infected cells (50, 51). Furthermore, TNFAIP3 was shown to promote gastric cancer cell proliferation, migration, and invasion by stabilizing Snail and ZEB1 proteins (52).

### Pancreatic cancer

YOD1 and OTUD7B were highly expressed in pancreatic cancer (PC) tissues and could propagate the proliferation and metastasis of PC cells (53, 54). Specifically, OTUD7B enhanced the EGFR and MAPK signaling pathways (54).

### Colorectal cancer

In colorectal cancer (CRC), OTUB1 and OTUD6A promoted tumor growth by stabilizing β-catenin (55) and Drp1 (56), respectively. However, silencing OTUD3 enhanced the proliferation and migration of CRC cells (57).

### Esophageal cancer

OTUB1 and OTUB2 potentiated esophageal squamous cell carcinoma (ESCC) proliferation and metastasis by regulating the stability of Snail and YAP1/TAZ proteins, respectively (58, 59).

### Other cancers

In various cancer types, OTU family members exhibit various oncogenic mechanisms. In brief, certain OTU members were oncogenic in specific cancers, such as OTUB1 in glioma (6), head and neck squamous cell carcinoma (HNSCC) (60), oral squamous cell carcinoma (OSCC) (61), and papillary thyroid carcinoma (PTC) (62); OTUB2 in intrahepatic cholangiocarcinoma (ICC) (63) and cervical cancer (64); OTUD4 in glioblastoma (GBM) (65); OTUD6A in prostate cancer (66); OTUD6B in laryngeal squamous cell carcinoma (67) and multiple myeloma (68); TNFAIP3 in glioma (69) and melanoma (70).

In contrast, other OTU members exert *anti*-cancer effects in specific cancers, such as YOD1 in HNSCC (71) and cervical cancer (72); OTUD1 and OTUD6B in clear cell renal cell carcinoma (ccRCC) (73, 74); TNFAIP3 in nasopharyngeal carcinoma (NPC) (75).

Overall, OTU family members manifest diverse and complex roles in cancer biology, offering promising targets for therapeutic strategies and deeper insights into cancer mechanisms.

## Dual role of OTUs in specific cancers

**Figure 4:**
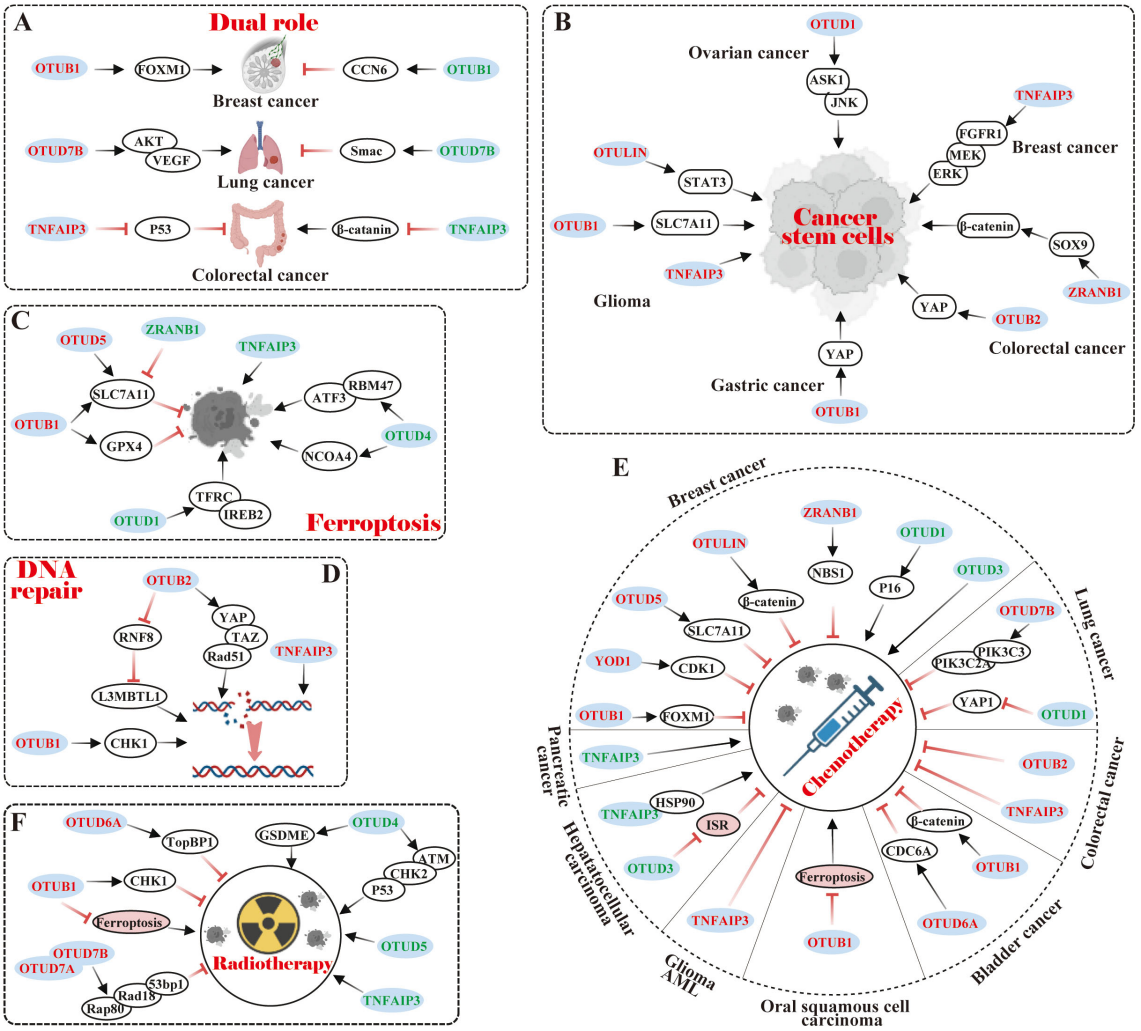
Relationship of OTU family members to tumor characteristics. **(A)** Dual role of OTUs in specific cancers. **(B)** OTUs maintain cancer stem cell-like properties. **(C)** OTUs regulate tumor ferroptosis. **(D)** OTUs participate in DNA repair. **(E, F)** OTUs are involved in tumor chemotherapy and radiotherapy. The red font represents OTUs exerting an oncogenic effect in specific cancers, while the green font is for an inhibitory effect.

The dual roles of OTU family members in specific cancers present intriguing mechanistic complexities, exemplified by OTUB1 in breast cancer, OTUD7B in lung cancer, and TNFAIP3 in CRC (**Figure 4A**).

Notably, OTUB1 exhibits context-dependent functional duality in breast cancer progression. Karunarathna U et al. (76) demonstrated that OTUB1 stabilized the oncogenic transcription factor FOXM1 by removing K48-Ub, thereby enhancing proliferation and epirubicin resistance in MCF-7 cells. Paradoxically, Zhao Y et al. (77) revealed that OTUB1 suppressed tumorigenic properties in 4T1 cells through a non-catalytic inhibition of CCN6 ubiquitination, ultimately impairing cell migration, proliferation, and viability. These opposing effects highlight the cell type-specific regulatory mechanisms of OTUB1 in breast cancer.

In lung cancer, OTUD7B displays multifaceted roles in lung cancer pathogenesis with divergent clinical implications. Pang Z et al. (78) correlated elevated OTUD7B expression with poor prognosis in lung adenocarcinoma tissues; Lin DD et al. (79) further showed that OTUD7B promoted *in vitro* proliferation and *in vivo* tumorigenicity of NCI-H358 cells via the AKT/VEGF pathway. In contrast, Sun C et al. (80) reported that OTUD7B exacerbated hyperthermia-induced cytotoxicity in A549 and CALU-3 cells by amplifying Smac-dependent mitochondrial dysfunction, while Zhang B et al. (81) identified its tumor-suppressive role in impeding LCL161-induced A549 and H1299 cells invasion and migration through TRAF3 deubiquitination-mediated NIK suppression. These findings suggest microenvironment-dependent functional switching of OTUD7B.

In CRC, TNFAIP3 exhibits paradoxical regulatory effects in colorectal carcinogenesis. Shao L (82) utilized TNFAIP3^fl/fl^ villin-Cre APC^min/+^ genetic mouse model to demonstrate its tumor-suppressive role in restricting Wnt signaling and suppressing colon tumorigenesis. Conversely, Liu J et al. (83) observed TNFAIP3 overexpression in human CRC tissues and adenomatous polyps, where it attenuated p53 expression in HEK293 cells, suggesting a potential oncogenic contribution to polyp malignancy. This duality underscores the need for context-specific evaluation of TNFAIP3 in CRC progression.

The observed functional duality of OTU family members in cancer pathogenesis arises from three interconnected mechanistic layers: substrate-specific modulation, signaling pathway plasticity, and therapeutic context dependency. Primarily driven by their context-dependent substrate engagement, OTUs can selectively stabilize oncoproteins (e.g., FOXM1) while destabilizing tumor suppressors (e.g., CCN6) through precise ubiquitin chain editing, creating opposing biological outcomes within the same cancer type. This functional dichotomy is further amplified by their ability to differentially regulate critical signaling hubs—such as simultaneously modulating NF-κB activation through TRAF3 deubiquitination while suppressing Wnt/β-catenin signaling via APC complex stabilization—effectively rewiring tumor cell fate decisions. Furthermore, OTUs establish dynamic feedback loops in drug response pathways, exemplified by their capacity to confer chemoresistance through drug target stabilization (e.g., epirubicin resistance via FOXM1 protection) while sensitizing cells to targeted therapies by enhancing pro-apoptotic signaling (e.g., Smac-mediated mitochondrial dysfunction). These multilayered regulatory mechanisms, operating through spatial-temporal control of ubiquitin code interpretation, ultimately dictate the paradoxical tumor-promoting versus tumor-suppressing phenotypes observed across cancer subtypes.

## OTUs in cancer therapy and patient prognosis

Currently, some OTUs have been reported to possess roles in preserving the cancer stemness and ferroptosis properties, which are intimately implicated not only in tumor aggressiveness and recurrence but also in the treatment and prognosis of patients.

### OTUs-mediated cancer stem cell properties

Cancer stem cells (CSCs) are characterized by their high metastatic potential, tumor initiation abilities, and capabilities for self-renewal, differentiation, and drug resistance traits similar to normal stem cells. Recent studies have emphasized the critical role that some OTU members play in maintaining CSC characteristics by regulating core transcription factors such as SLC7A1, YAP, ASK1, and SOX9 (**Figure 4B**).

Specifically, OTUB1 stimulated glioma cell stemness by stabilizing the SLC7A11 protein to suppress ferroptosis (84). Additionally, OTUB1 and OTUB2 served as pivotal components in maintaining cancer stemness and promoting metastasis via deubiquitination and stabilization of YAP proteins in gastric and colon cancer, respectively (85, 86). Multi-omics screening has identified that OTUD1 stabilized ASK1 by recruiting it in a deubiquitinase-independent manner, which activated the downstream JNK signaling pathway to maintain ovarian cancer stem cells (87). Interestingly, ALDH-positive breast cancer stem cells (BCSCs) were reduced in TNFAIP3 knockout in BCSCs, possibly since TNFAIP3 facilitated the growth of ALDH-positive BCSCs in part through the FGFR1/MEK/ERK pathway (88). ZRANB1 modulated SOX9 stability in CRC cells by diminishing its ubiquitination, which in turn potentiated the SOX9-mediated USP22/Wnt/β-catenin pathway to uphold CRC stemness characteristics (89). In GBM, TNFAIP3 and OTULIN maintained the stemness and self-renewal capacity of GBM stem-like cells (GSCs) (90, 91). Specifically, preferential expression of OTULIN in GSCs restricted linear ubiquitination on STAT3 and drove persistent STAT3 signaling (91).

Overall, OTU members have been shown to provide a critical role in the sustainment of CSC properties via the regulation of cellular signaling pathways and core transcription factors. Several studies have illustrated that CSC properties can significantly confer drug resistance in cancer cells, which seriously affects tumor response to treatment and patient prognosis. Thus, the OTU family may hold a pivotal position in chemo- or radiotherapy resistance in tumors.

### OTUs in Ferroptosis

Ferroptosis is a type of programmed cell death, distinct from apoptosis and necrosis, characterized by iron-dependent accumulation of lipid peroxides. Research has shown that in cancer, ferroptosis can inhibit the growth of tumor cells. Therefore, inducing ferroptosis in cancer cells may be a novel *anti*-cancer therapeutic strategy, and OTUs influence tumor progression by modulating the stability of ferroptosis-related proteins (**Figure 4C**).

SLC7A11, also known as xCT, is a transporter protein that influences glutathione (GSH) synthesis, a vital *anti*-oxidant, by regulating intracellular cysteine levels. In many malignancies, upregulation of SLC7A11 expression contributes to tumor cell resistance to ferroptosis, which promotes tumor growth, metastasis, and drug resistance. Currently, several OTU family members, such as OTUB1 (84, 92) and OTUD5 (93), can positively regulate the protein stability of SLC7A11, thereby inhibiting ferroptosis. Interestingly, the deubiquitinase ZRANB1 was identified as the E3 ligase of SLC7A11 to degrade it, which suppressed GSH synthesis and led to lipid peroxidation and elevated ferroptosis (94).

In addition to that, OTUs can govern ferroptosis in tumor cells via other pathways. For instance, OTUB1 inhibited ferroptosis by improving GPX4 protein stability and reducing intracellular reactive oxygen species (ROS), which in turn promoted gastric cancer metastasis (95). In contrast, OTUD1, OTUD4, and TNFAIP3 could induce the genesis of ferroptosis. Specifically, OTUD1 facilitated TFRC-mediated iron transport via deubiquitination and stabilization of IREB2, causing ROS production and increased ferroptosis in CRC (96). OTUD4 potentiated ferroptosis in ICC and ccRCC by regulating the stability of NCOA4 protein and RBM47/ATF3 axis, respectively (97, 98). In A549 lung cancer cells, TNFAIP3 exhibited a significant induction of ferroptosis (99).

In short, ferroptosis represents a promising prospect for extensive research in cancer therapy, but additional clinical trials and studies are necessary to gain insight into its mechanisms and applications.

### OTUs in DNA repair

DNA repair is a fundamental cellular process aimed at restoring damaged DNA to its normal state. However, cancer cells often exploit this mechanism to survive chemotherapy and thus evade treatment. Recent studies have emphasized the critical role of several OTU members in DNA repair (**Figure 3D**). In lung cancer, for example, OTUB1 stabilized the CHK1 protein through deubiquitination, enhancing the cell’s ability to repair DNA and aiding cancer cell survival (100). In endometrial cancer, OTUB2 promoted Rad51 expression via the YAP/TAZ pathway, supporting homologous recombination repair and protecting cells from drugs like cisplatin (101). Additionally, OTUB2 regulated L3MBTL1 at DNA break sites by counteracting RNF8, optimizing DNA repair pathways (102). TNFAIP3, which was highly expressed in invasive breast cancer, increased the efficiency of error-free DNA homologous recombination and diminished error-prone non-homologous DNA end-joining, which stabilized the genome and conferred resistance to DNA damage (103). These data indicate that OTUs exert effects on DNA repair by regulating protein stability and influencing the choice of repair mechanism, etc.

### OTUs in chemotherapy

Chemotherapy attacks cancer cells by interfering with their DNA replication, repair, and cell division. Many studies have pointed out that the expression level of OTUs was related to the efficacy of chemotherapy. Comparatively, it was identified that the majority of OTUs conferred chemoresistance to tumor cells, while a small percentage of OTUs sensitized tumor cells to chemotherapy (**Figure 4E**).

Currently, members of OTUs were reported to both promote resistance and sensitization in specific cancers. In breast cancer, for instance, OTUB1, YOD1, OTUD5, OTULIN, TNFAIP3, and ZRANB1 drove chemoresistance, whereas OTUD1 and OTUD3 were sensitized to chemotherapy. Specifically, OTUB1 deubiquitinated and stabilized FOXM1, thereby conferring epirubicin resistance (76). YOD1 positively regulated CDK1 stability and drove cisplatin and paclitaxel resistance (20). OTUD5 inhibited ferroptosis by stabilizing SLC7A11, thereby diminishing paclitaxel susceptibility (104). OTULIN enhanced doxorubicin resistance by activating the Wnt/β-catenin pathway (105, 106).TNFAIP3 could confer tamoxifen resistance (107). ZRANB1 was implicated in radioresistance and PARPi resistance, such as olaparib and tarazoparib, by modulating the stability of NBS1 and upregulation of the MRN complex (108). On the contrary, OTUD1 rendered TNBC cells sensitive to doxorubicin by up-regulating P16 expression (109). OTUD3 overexpression significantly enhanced the responsiveness of MCF-7 cells to paclitaxel (110). In lung cancer, OTUD7B facilitated osimertinib resistance in lung adenocarcinoma cells through PIK3C3 stabilization and PIK3C2A transcription (111). However, OTUD1 conferred erlotinib susceptibility in NSCLC by repressing the nuclear translocation of YAP1 (112).OTUD5 knockdown also potentiated resistance to doxorubicin and cisplatin in NSCLC cells (113).

Members of OTUs were also reported to confer chemotherapy resistance in specific cancers. In CRC, for example, OTUB2 and TNFAIP3 confer cisplatin resistance to CRC cells (114, 115). In BLCA, OTUB1 and OTUD6A potentiated BLCA resistance to cisplatin and gemcitabine by deubiquitinating and stabilizingβ-catenin and CDC6, respectively (116, 117). In OSCC, OTUB1 induced cisplatin resistance by suppressing ferroptosis (118). Furthermore, TNFAIP3 enhanced the resistance of GBM and acute myeloid leukemia (AML) cells to O6 alkylating agents and daunorubicin, respectively (119, 120).

Surely, OTUs were reported to be sensitized to chemotherapy in specific cancers. Such as in HCC, OTUD3 and TNFAIP3 rendered HCC cells responsive to sorafenib by antagonizing the integrative stress response (ISR) and binding to HSP90, respectively (121, 122). In SW1990 pancreatic cancer cells, overexpression of TNFAIP3 increased chemosensitivity to gemcitabine (123).

In view of the fact that many OTUs confer chemoresistance to tumor cells, the future is dedicated to the study of OTU inhibitors as candidates for targeted therapy. Meanwhile, combining OTU inhibitors with other means, such as radiotherapy and immunotherapy, may improve therapeutic efficacy.

### OTUs in radiotherapy

Radiotherapy suppresses the growth of cancer cells by directly destroying their DNA with high-energy radiation, which is mainly applied for localized tumor control. OTUs impact the efficacy of radiotherapy by regulating cellular response mechanisms, DNA repair and ferroptosis, and other mechanisms (**Figure 4F**).

Currently, OTUB1, OTUD6A, and OTUD7B were reported to be engaged in resistance to radiotherapy. Mechanistically, OTUB1 inhibited radiation-induced cellular ferroptosis, which triggered radiotherapy resistance in NPC (124). Furthermore, OTUB1 and OTUD6A deubiquitinated and stabilized CHK1 and TopBP1, which regulated DNA damage and repair and promoted radiation resistance in lung and breast cancer, respectively (21, 100). Analogously, OTUD7A interacted with OTUD7B to promote OTUD7B recruitment of Rap80/BRCA1-A, Rad18, and 53bp1, which enhanced cellular resistance to ionizing radiation, and DNA damage repair (125).

On the contrary, high expression of OTUD4, OTUD5, and TNFAIP3 could enhance the sensitivity of cancer cells to radiotherapy. Specifically, OTUD4 sensitized NSCLC cells to radiotherapy through ATM/CHK2/P53 signaling and suppressed homology-directed repair of ionizing radiation-induced DNA double-strand breaks (126). OTUD4-mediated GSDME deubiquitination also enhanced radiosensitivity in NPC by inducing pyroptosis (127). Furthermore, OTUD5 overexpression enhanced the sensitivity of cervical cancer cells to radiotherapy (128), whereas TNFAIP3 knockdown decreased the sensitivity of NPC cells to radiotherapy (129).

Collectively, OTUs influence the efficacy of radiotherapy through a variety of mechanisms, emerging as valuable targets in cancer research and treatment. Inhibitors or agonists targeting OTUs can potentially improve the efficacy of radiotherapy in specific cancers.

### Clinical significance of OTUs

Numerous studies have shown that members of the OTU family exhibited significant differences in clinical expression between cancerous tissues and normal tissues. The expression levels of some members were associated with TNM stage or lymph node metastasis or shortened overall survival in cancer patients, indicating a poor prognosis. For instance, OTUB1 in glioma (6), CRC (130), GC (131), and HCC (132); OTUB2 in BC (133); OTUD2 in NSCLC (134); TNFAIP3 in BC (135, 136) and ESCC (137).

In contrast, other OTU family members were positively observed to be linked to patients’ overall and disease-free survival. In HCC, for example, decreased OTUD7B expression was related to increased tumor volume, presence of satellite nodules, vascular invasion, and early recurrence (138, 139). In pancreatic ductal adenocarcinoma (PDAC), TNFAIP3 expression was positively correlated with tumor differentiation, TNM stage, and patient survival, suggesting a potential *anti*-cancer role (140). Reduced expression of ZRANB1, a favorable factor, in HCC tissues and cell lines, facilitated tumor recurrence and metastasis (141).

**Figure 5:**
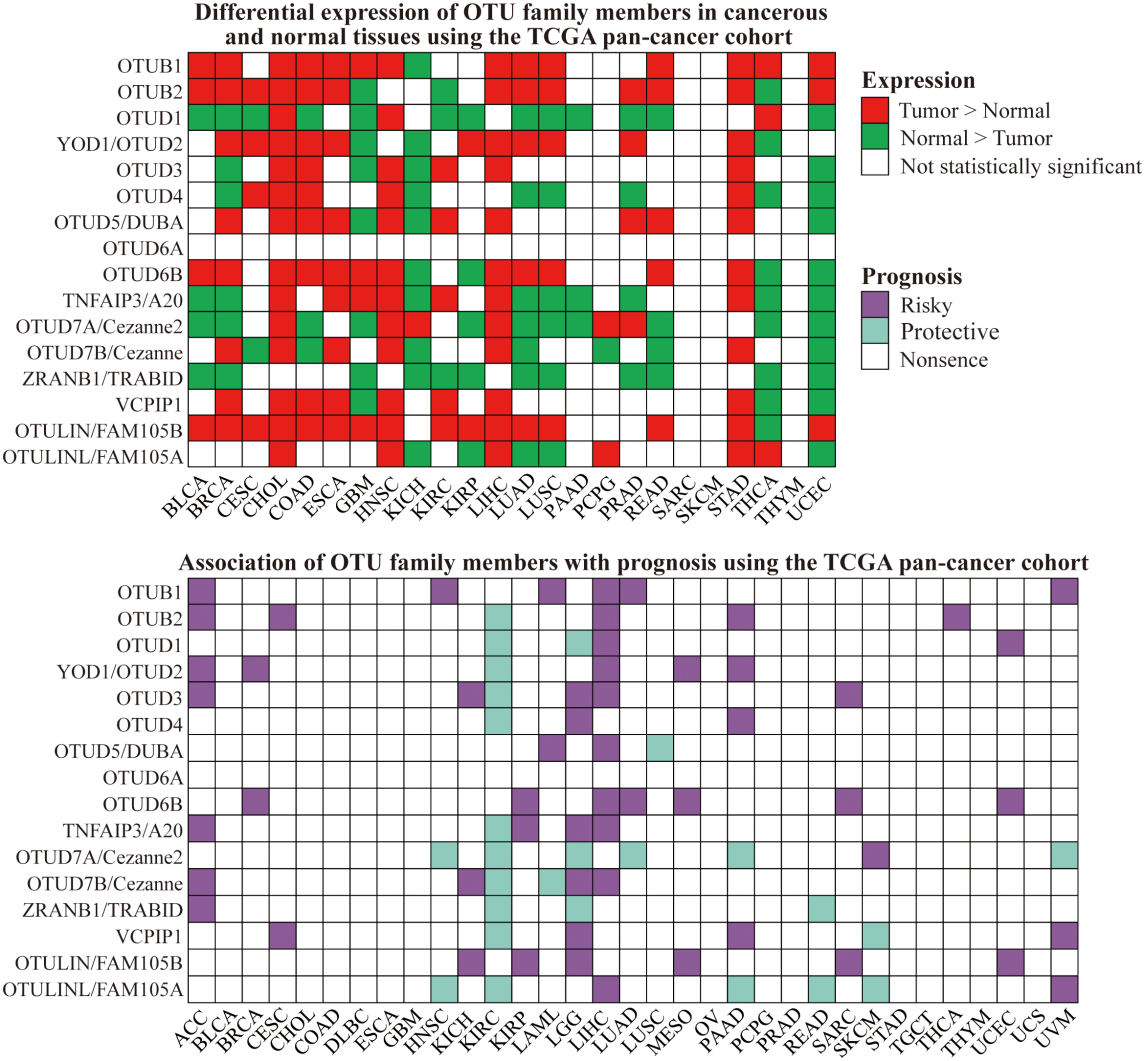
Differential expression and prognostic value of OTU family members in cancer and normal tissues using the TCGA pan-cancer cohort.

To broaden our understanding of the expression patterns of OTU family members in tumors and their association with patient prognosis, we conducted a pan-cancer analysis utilizing The Cancer Genome Atlas (TCGA) database, providing a comprehensive landscape of OTUs’ expression and prognostic relevance (**Figure 5**). Through these studies, we can notice that the expression patterns of OTUs family proteins in various cancers are closely related to the biological behaviors of tumors and the clinical prognosis of patients, providing important biomarkers for cancer diagnosis, treatment, and prognostic assessment.

## OTUs participate in the host immune response

**Figure 6:**
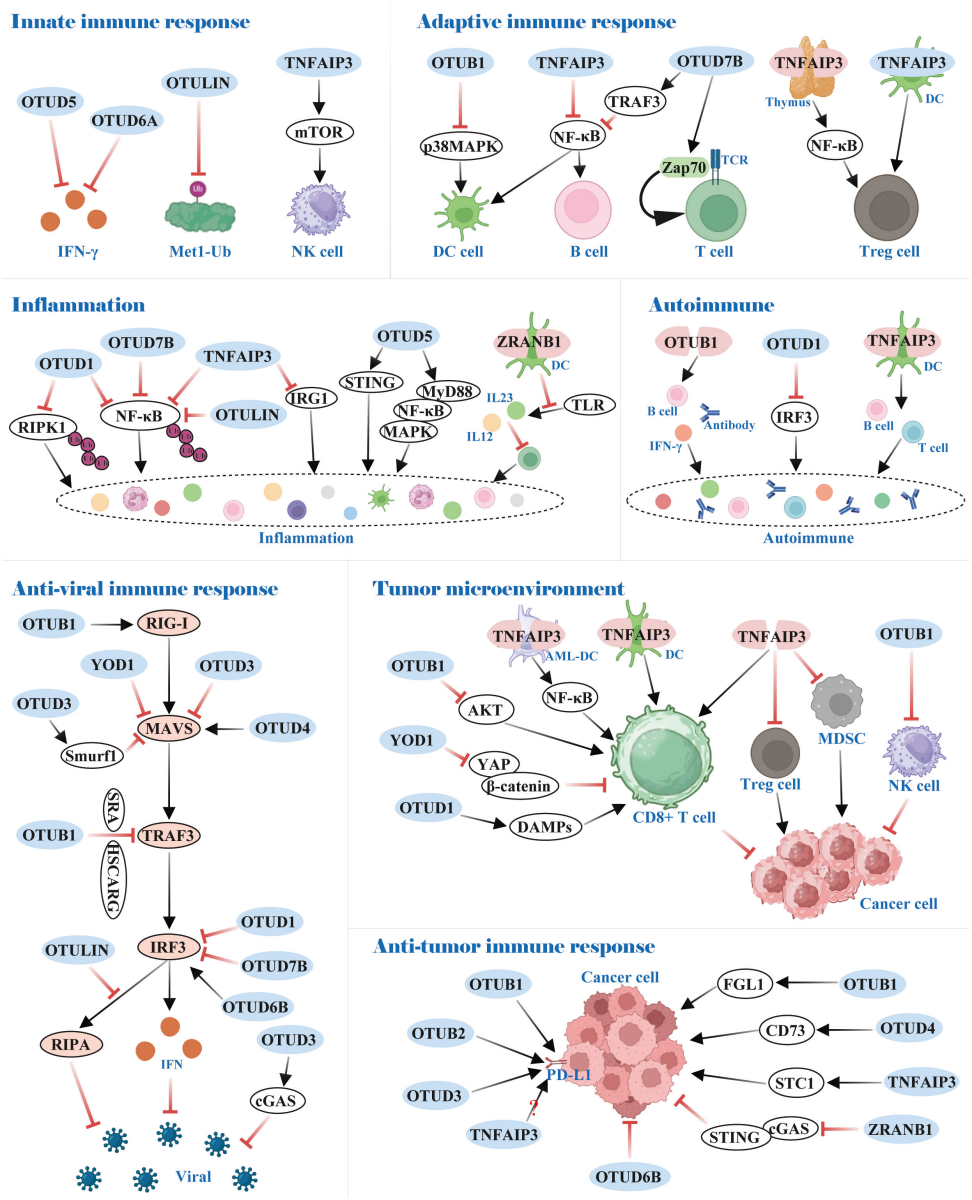
Functions of OTUs in immunity. OTUs are involved in innate and adaptive immune responses, inflammation and autoimmunity, and *anti*-viral and *anti*-tumor immune responses.

Recent studies have shown that the role of the OTUs family of proteins in the body’s immune system has received increasing attention. For instance, certain OTUs possess crucial roles in the differentiation, proliferation, and functional maintenance of T and B cells. Moreover, they can also influence the inflammatory response and *anti*-viral immune response by governing the NF-κB and interferon pathways, respectively (**Figure 6**).

### OTUs participate in the body’s immune response by regulating the differentiation and development of immune cells

The innate immune system serves as the body’s rapid first-line defense against invading pathogens, with type I interferon (IFN-I) production constituting a central protective mechanism in this initial response. Research has identified OTUD5 and OTUD6A as potent suppressors of IFN-I generation, establishing their roles as negative regulators of innate immunity (142, 143). Notably, while Met1-linked ubiquitin chains (M1-Ub) were crucial for amplifying innate immune signaling, the deubiquitinating enzyme OTULIN counteracted this process by selectively dismantling M1-Ub chains, thereby restraining excessive inflammatory activation (144).

OTUs also maintain the homeostasis of a portion of innate immune cells. In NK cells, for example, TNFAIP3 controlled NK cell homeostasis by regulating mTOR activity to prevent its spontaneous death (145). Dendritic cells (DCs), an antigen-presenting cell (APC), link innate and adaptive immune responses. OTUB1 and TNFAIP3 restrained p38MAPK and NF-κB activation, respectively, which prevented DCs hyper-activation under homeostatic conditions (146–148).

In adaptive immunity, OTUs mediate the stability and activity of key proteins linked to immune cells by removing the ubiquitin chain. In B cells, for instance, OTUD7B deubiquitinated TRAF3 and prevented its degradation, thereby hindering aberrant nonclassical NF-κB activation (149). Also, TNFAIP3 was reported to be a negative regulator of NF-κB signaling, which impeded B cells’ hyper-activation to maintain immune homeostasis (150). Importantly, OTUD7B promoted T cell activation by deubiquitinating and activating Zap70, a central mediator of TCR proximal signaling (151). Regulatory T cells (Treg), essential for maintaining immune tolerance, are also activated by NF-κB transcription factor. In the thymus, TNFAIP3 deficiency dominated differentiation over Treg cells owing to enhanced NF-κB activation, but silencing TNFAIP3 in DCs dampened Treg cells activation (152, 153). Conversely, TNFAIP3 overexpression in DCs led to the development of tolerogenic DCs, which facilitated the induction of Treg cells (154). All of these provide a promising potential in the treatment of inflammatory and autoimmune diseases.

### OTUs-mediated inflammatory response and autoimmunity

Investigations have indicated that distinct OTUs family proteins exert either *anti*-inflammatory or pro-inflammatory responses, e.g., OTUD1, OTUD7B, OTULIN, and TNFAIP3 function as *anti*-inflammatory, whereas the contrary is true for OTUD5 and ZRANB1.

In OTUD1^-/-^ mice, inflammation was augmented in models of inflammatory bowel disease, acute hepatitis, and sepsis. Mechanistically, OTUD1 inhibited inflammation by cleaving K63-Ub from RIPK1 and NF-κB, respectively, thereby dampening NF-κB signaling pathway transduction (155, 156). Analogously, OTUD7B and OTULIN attenuated NF-κB activation by selectively removing K63-Ub and M1-Ub on NF-κB, respectively, thereby inhibiting inflammation induced by TNF receptor (TNFR) signaling (157–159). TNFAIP3, also a negative regulator of NF-κB signaling, predominantly used its zinc finger structural domain 7 (ZF7) to curb inflammatory signaling. Additionally, TNFAIP3 negatively regulated immune response gene 1 (IRG1) expression at the transcriptional level (160, 161).

In radiation pneumonitis (RIP), OTUD5 upregulated by USP11 acclimated endothelial cell inflammation through the STING signaling pathway (162). In inflammatory bowel disease, OTUD5 was significantly overexpressed and increased TNF-α release (163). Mechanistically, OTUD5 interacted with MyD88 and cleaved its K11-Ub, which enhanced MyD88 oligomerization and subsequently promoted Myddosome formation, activation of NF-κB and MAPK signaling, and inflammatory cytokine production (164). Furthermore, ZRANB1 deletion in DCs inhibited Toll-like receptor (TLR)-induced expression of IL12 and IL23, which impaired inflammatory T cell differentiation and protected mice from autoimmune inflammation (165).

Consistent with the aforementioned results, OTUB1, OTUD1, and TNFAIP3 restricted the development of autoimmune diseases through *anti*-inflammatory responses. Specifically, OTUB1 deficiency exhibited an aberrantly activated phenotype in B cells, causing B cell proliferation, antibody, and IL-6 hyper-production, and lupus-like autoimmunity (166). OTUD1 prevented excessive interferon production-induced autoimmune disease by removing K63-Ub on Lys98 of IRF3, thereby dampening IRF3 nuclear translocation and transcriptional activity, which blocked RIG-I-like receptor signaling (167). Furthermore, TNFAIP3 ablation in DCs altered T-cell and B-cell homeostasis, which primarily promoted the progression of autoimmune liver disease (168).

### OTUs-mediated anti-viral immune response

RIG-I-like receptor (RLR) pathway activates downstream signaling primarily through recognition of viral RNA, which, involves the participation of multiple molecules including, but not limited to, RIG-I, MAVS, TRAF3, and IRF3, and ultimately facilitates interferon-mediated *anti*-viral immune responses. OTUs, as deubiquitinases, regulate these pivotal molecules in post-transcriptional modifications, thereby impacting on the organism’s *anti*-viral immunity.

RIG-I is an intracellular pattern recognition receptor that recognizes viral 5’triphosphate RNA and double-stranded RNA. During influenza A virus (IAV) infection, OTUB1 activated RIG-I via a dual mechanism of K48-Ub hydrolysis and formation of an E2-repressive complex with UBCH5c, which stimulated the RIG-I signaling cascade and the *anti*-viral response (169).

The adaptor protein MAVS binds to activated RIG-I and further triggers a series of signal transduction events, such as recruitment of the TRAF family and activation of the transcription factor IRF3. YOD1 and OTUD3 abrogated the formation of prion-like aggregates of MAVS by interacting with MAVS and cleaving K63-Ub, leading to attenuation of IRF3 and IFN-β production (170, 171). Additionally, OTUD1 up-regulated E3 ubiquitin ligase Smurf1 expression via deubiquitination, which increased the degradation of MAVS and the MAVS/TRAF3/TRAF6 signalosome (172, 173). Yet, viral infection induced an IRF3/7-dependent up-regulation of OTUD4, which bound to MAVS to remove K48-Ub, thereby maintaining MAVS stability and promoting innate *anti*-viral signaling (174).

TRAF3 interacts with MAVS, contributing to signaling and enhancing the downstream *anti*-viral response. OTUB1 was recruited to TRAF3 by Scavenger receptor A (SRA) and HSCARG to negatively regulate its protein stability, which counterbalanced *anti*-viral innate immunity (175, 176).

IRF3 was identified as a key regulator of interferon production. Currently, several OTU proteins have been reported to negatively regulate the protein abundance of IRF3, including OTUB1 (177), OTUD1 (178), OTUD7B (179), OTULIN (180), and TNFAIP3 (181, 182). Mechanistically, OTUD1 caused the dissociation of IRF3 from the promoter region of the target gene by cleaving K6-Ub from IRF3, without disturbing its protein stability, dimerization, and nuclear translocation (178). In addition, OTUD7B promoted the degradation of IRF3 by removing K63-Ub at the IRF3 Lys7 residue (179). Interestingly, IRF3 activates another interferon-independent *anti*-viral pathway termed the RIG-I-induced apoptotic pathway (RIPA). OTULIN suppressed RIPA by deubiquitinating IRF3 to prevent its mitochondrial translocation (180). Nevertheless, OTUD6B positively modulated the IRF3-mediated *anti*-viral immune response by stabilizing IRF3 protein abundance via hydrolysis of K33-Ub on IRF3 Lys315 residue (183).

In addition, cyclic GMP-AMP synthase (cGAS) behaves as a major DNA sensor and initiates DNA-stimulated innate immune responses. OTUD3 was reported to stabilize and potentiate cGAS enzyme activity, which facilitated the *anti*-DNA viral immune response (184).

The RLR pathway initiates antiviral immunity by detecting viral RNA through a RIG-I/MAVS/TRAF3/IRF3 signaling cascade to drive interferon production, while OTUs dynamically fine-tune this response via spatiotemporal regulation of post-translational modifications. These enzymes establish bidirectional control networks across RLR signaling nodes through targeted deubiquitination, providing molecular precision for antiviral immune modulation. Furthermore, OTUs play dual roles in antibacterial immunity, as structurally and pharmacologically elucidated by Dirk Schlüter et al. (185). Pathogens evade immune surveillance by secreting OTU-mimicking effector proteins that hijack the host ubiquitination system through specific ubiquitin chain hydrolysis, thereby suppressing NF-κB-mediated inflammatory responses and attenuating chemokine or cytokine storms. Conversely, host OTUs like TNFAIP3 maintain immune homeostasis via dynamic regulation of signaling pathways, particularly through negative feedback modulation of the NF-κB cascade to balance protective immunity and inflammatory control.

## Role of OTUs in the tumor microenvironment and immune escape

The tumor microenvironment (TME) describes the sophisticated landscape of cells, signaling molecules, and stroma surrounding tumor cells, which contributes to tumourigenesis, progression, and therapeutic response. As deubiquitinases, OTU family proteins exert an influential role in tumor progression and immune escape by regulating intracellular ubiquitination levels, which affects the tumor microenvironment and immune cell function.

### OUTs-mediated anti-tumor immunity in TME

In TME, partial OTUs attenuated *anti*-tumor immunity by dampening CD8^+^ T-cell function. For instance, OTUB1 was recruited to the membrane mediated by IL-15, which restrained the ubiquitin-dependent activation of AKT and thus inhibited the activation of CD8^+^ T and NK cells (186). In the B16 mouse melanoma tumor model, TNFAIP3 silencing in DCs resulted in an augmented amount of tumor-specific cytotoxic T cells (187); in AML, TNFAIP3 depletion in AML-DCs potentiated autologous cytolysing T cell (CTL)-specific killing of progenitor AML cells via the NF-κB pathway (188). Likewise, adoptively transferred *anti*-tumor CD8^+^ T cells harboring a deletion of TNFAIP3 enhanced IFN-γ and TNF-α production and reduced PD-1 expression, which exhibited superior *anti*-tumor activity *in vivo* (189, 190).

On the contrary, YOD1 and OTUD1 positively regulated CD8^+^ T cells. Specifically, YOD1 prevented CD8^+^ T cell exhaustion by inactivating the YAP/β-catenin pathway in HCC (191). OTUD1 facilitated the release of damage-associated molecular patterns (DAMPs), which in turn recruited tumor-responsive T cells and curbed colon cancer progression (96).

Tregs, tumor-associated macrophages (TAMs), and DCs collectively form a dynamic immunosuppressive network within the TME, collaboratively driving tumor immune evasion. Although the mechanistic roles of these cells have been partially elucidated, the regulatory functions of OTUs in modulating their activities remain underexplored. For instance, recent studies demonstrate that TNFAIP3 silencing dampened infiltration of Treg cells and myeloid-derived suppressor cells (MDSCs), while the numbers of dendritic cells and macrophages remained unaffected (187, 192). This finding underscores the potential therapeutic value of targeting OTUs to remodel the immunosuppressive TME.

Overall, OTUs serve a complicated role in TME, and further investigations could provide insights into their functions and mechanisms, thus shedding new light on tumor prevention and treatment.

### OTUs influence the efficacy of immunotherapy via the regulation of immune checkpoints

Immune checkpoints work as regulators of the immune system, and immunotherapy can be applied to combat cancer by strengthening the immune response through the regulation of these immune checkpoints.

PD-L1, a crucial immune checkpoint protein, is up-regulated in tumor cells to evade surveillance by the immune system, preventing T cells from effectively recognizing and attacking the tumor. Currently, certain OTUs stabilize PD-L1 expression to propagate tumor immune escape. In the A549 lung cancer and 4T1 breast cancer mouse models, OTUB1 knockdown significantly enhanced the *anti*-tumor immune response of mice. Mechanistically, OTUB1 attenuated tumor immunity in NSCLC and breast cancer by cleaving the K48-Ub of PD-L1 to prevent degradation of PD-L1 via the ERAD pathway (193, 194). Consistent with this, analogous phenomena were observed in OTUB2 in NSCLC and OTUD3 in diffuse large B-cell lymphoma (DLBCL) (195, 196). Nevertheless, TNFAIP3 was controversial in regulating PD-L1 expression. Guo W et al. (197) suggested that TNFAIP3 knockdown significantly down-regulated PD-L1 expression in melanoma A375 and A2958 cells. However, Zou J et al. (198) noted that as the concentration of TNFAIP3 plasmid transfected in MDA-MB231 and A375 cells increased, the abundance of PD-L1 decreased. Breitenecker K et al. (199) also showed that PD-L1 was highly expressed in TNFAIP3 knockout in lung adenocarcinomas. Further investigations are required concerning the role of TNFAIP3 in regulating the expression of PD-L1.

In addition to their core regulatory functions, various OTUs participate in tumor immune regulation by specifically modulating key immune checkpoint molecules. Specifically, OTUD1 enhanced FGL1 stability through deubiquitination, which mediated immune escape and progression of metastatic colorectal cancer (200). OTUD4 deubiquitinated CD73 to offset the ubiquitination of TRIM21, causing CD73 stabilization to suppress the immune response in breast cancer (201). TNFAIP3 suppressed STC1 phosphorylation at Thr86 by GSK3β to alleviate STC1 protein degradation, which promoted immune evasion in colorectal cancer (202). ZRANB1 could stabilize the entire chromosome, and its inhibition prevented autophagic degradation of cGAS to further stimulate the cGAS/STING innate immune pathway. ZRANB1 inhibition facilitated *anti*-tumor immune surveillance and was sensitive to *anti*-PD-1 therapy (203). Notably, these regulatory mechanisms provide novel directions for developing combination immunotherapy strategies targeting OTUs.

The OTU family proteins profoundly shape the tumor immune microenvironment through diverse molecular mechanisms, offering novel targeted strategies to enhance the efficacy of immune checkpoint inhibitors. For example, in DLBCL, the OTUD3 inhibitor Rupatadine competitively bond to OTUD3 to block PD-L1 deubiquitination, promoting its proteasomal degradation, thereby alleviating PD-1/PD-L1-mediated immune suppression and enhancing *anti*-tumor T-cell activity (196). In TNBC, ST80 disrupted OTUD4-dependent stabilization of CD73, reducing immunosuppressive adenosine levels, reactivating CD8^+^ T-cell function, and reversing resistance to *anti*-PD-L1 therapy (201). Additionally, in ESCC, all-trans retinoic acid (ATRA) suppressed the Snail signaling pathway by inducing OTUD6B expression, synergistically improving responses to *anti*-PD-1 therapy (204). Collectively, these studies highlight the potential of OTU family members as “immune regulatory hubs”. Targeted modulation of specific OTU proteins may achieve multi-layered synergies with existing immunotherapies, though further elucidation of their substrate specificity and dynamic regulatory networks is required to mitigate potential antagonistic risks.

## Directions and challenges for future research

The OTU family deubiquitinases play pivotal roles in tumorigenesis, immune evasion, and tumor microenvironment remodeling by regulating ubiquitination modification networks. Recent studies have uncovered their diverse functions in tumor proliferation, metastasis, and therapy resistance. For instance, OTUB1 promoted NPC progression by regulating ferroptosis and radioresistance (124), while OTUD3 and OTUD4 impaired *anti*-tumor immune responses by stabilizing PD-L1 and CD73, respectively (196, 201). These findings position OTU family members as emerging therapeutic targets, yet their functional complexity and clinical translation challenges require systematic resolution.

Modern cancer research demonstrates an integrated innovation chain spanning from molecular mechanisms to clinical translation in the context of interdisciplinary convergence. At the molecular regulatory level, functional exploration of the OTU deubiquitinase family has opened new avenues for targeted therapies. For example, OTUB1 has emerged as a hotspot for small-molecule inhibitor design through its Asn45/Arg86 hydrogen-bond network (205), while the OTUD3 inhibitor OTUDin3 significantly suppresses NSCLC progression by targeting the S1 ubiquitin-binding site (206). Epigenetic regulation mechanisms, such as FTO-mediated m6A modification, further reveal OTUB1’s critical role in conferring radiotherapy resistance in NPC (124). These discoveries are driving the integration of single-cell multi-omics technologies with spatial metabolomic imaging to map the dynamic regulatory landscape of OTU enzymes within tumor immune-metabolic networks.

In therapeutic strategy development, structure biology-based precision designs (e.g., PROTAC degraders) and combination therapies—such as ST80 enhancing TNBC immunotherapy by disrupting the OTUD4-CD73 complex (201) and crizotinib suppressing NSCLC via targeting the OTUB1/pSTAT3 axis (207)—are overcoming functional redundancy challenges, while the combined use of ERRα inhibitors and metabolic modulators underscores the necessity of multi-target interventions (208). Concurrently, diagnostic and therapeutic innovations continue to advance. For instance, tumor-associated macrophages (TAMs) are associated with distant tumor metastasis and poor prognosis (209), while dynamic imaging of TAMs based on a two-step click chemistry protocol enables real-time visualization of breast cancer progression (210), while copper-based nanomaterials integrate diagnostic functions with photothermal/electromagnetic synergistic therapeutic capabilities through heterostructure doping (211, 212). Additionally, low-frequency rotating magnetic fields significantly inhibit breast cancer metastasis by disrupting F-actin polymerization (213). These technologies synergize with physical microenvironment modulation tools like ionizing radiation and ultrasound, forming a three-dimensional therapeutic network.

At the clinical translation level, biomarker stratification (e.g., the FTO-OTUB1 axis in NPC prognosis) and adaptive clinical trial designs (e.g., basket trials for OTUD3-high DLBCL) may improve precision. Dynamic monitoring techniques (e.g., ctDNA analysis) enable real-time tracking of therapeutic responses and compensatory pathway activation. However, vigilance is required regarding potential immune homeostasis disruption risks associated with OTU-targeted therapies. The deep integration of traditional medicine and modern technology is exemplified by the synergistic interplay between herbal formulations—which exert systemic regulatory effects in cancer management—and mesenchymal stem cell-derived exosomes that modulate mitochondrial gene functions (214–216). These approaches, combined with exercise-induced tumor immune microenvironment reprogramming and immunotherapy, establish a multi-tiered defense framework (217). Artificial intelligence further enriches early screening by uncovering associations between craniofacial genes and cancer susceptibility (218). Current research is advancing toward magnetic field-nanomaterial synergy platforms and imaging-guided dynamic monitoring systems, with a focus on integrating physical interventions, metabolic reprogramming, and gene-editing technologies to achieve a closed-loop precision therapy spanning OTU molecular network regulation to systemic immune activation. This evolution marks a comprehensive transformation in oncology toward a spatiotemporal dynamic regulation paradigm.

In conclusion, OTU family research is shifting from single-target exploration to multidimensional network analysis. By integrating structural biology, computational modeling, and clinical big data, future research may develop OTU-based molecular subtyping strategies for personalized treatment, synergizing with existing immunotherapies. However, overcoming functional complexity, optimizing inhibitor specificity, and ensuring clinical safety remain pivotal for translational breakthroughs. Advances in this field could redefine the landscape of cancer immunotherapy, offering novel strategies to improve patient outcomes.

## Conclusions

Collectively, OTU family members performs critical roles in tumorigenesis and immunomodulation. In the present review, we illustrated that OTUs can modulate multiple signaling pathways through deubiquitination, thereby affecting tumor growth, migration, and apoptosis. Alternatively, OTUs were able to influence the intensity and duration of the immune response and promote tumorigenesis and progression in the TME by regulating the inflammatory response and immune escape mechanisms. Hence, an in-depth investigation of the specific mechanisms of the OTU family and their roles in tumor and immunity will be significant for the development of novel *anti*-tumor therapeutic strategies.
